# Current Melanoma Treatments: Where Do We Stand?

**DOI:** 10.3390/cancers13020221

**Published:** 2021-01-09

**Authors:** Alvaro Moreira, Lucie Heinzerling, Nina Bhardwaj, Philip Friedlander

**Affiliations:** 1 The Tisch Cancer Institute, Icahn School of Medicine at Mount Sinai, New York, NY 10029, USA; nina.bhardwaj@mssm.edu (N.B.); philip.friedlander@mssm.edu (P.F.); 2 The Kimberly and Eric J. Waldman Department of Dermatology at Mount Sinai, New York, NY 10029, USA; 3 Department of Dermatology and Allergology, Ludwig-Maximilian University, Geschwister-Scholl-Platz 1, 80539 München, Germany; Lucie.Heinzerling@med.uni-muenchen.de

**Keywords:** melanoma, clinical trials, metastatic, adjuvant, neoadjuvant, resistance, toxicity

## Abstract

**Simple Summary:**

In this manuscript, we discuss recent updates on melanoma-related clinical trial data. We explore diverse topics, including new therapeutic agents and novel combinations that are being tested in early-phase clinical trials. Furthermore, we review long-term efficacy and safety data from the clinical trials that led to the currently approved drugs in the melanoma landscape. We analyze data from human clinical trials in the metastatic setting, adjuvant setting and neoadjuvant setting. Moreover, we review recent breakthroughs related to the management after resistance, as well as the discovery of new targets. Lastly, we explore clinical trials for non-cutaneous melanoma, such as uveal and mucosal melanoma.

**Abstract:**

Groundbreaking research in immunology and cancer biology in the last few decades has led to the discovery and development of novel therapeutics, such as immune checkpoint inhibitors and targeted therapies, which have revolutionized the clinical care of patients with metastatic melanoma. Updated data from the largest clinical trials continue to support the use of these treatment modalities, both in the metastatic and in adjuvant settings, with studies showing the predicted plateau effect on survival curves. However, with growing evidence that neoadjuvant therapy is also associated with high rates of recurrence-free survival, the question about whether patients should receive adjuvant or neoadjuvant treatment raises new questions about therapeutic options. Finally, management after resistance and intervention with novel immunotherapies are newer challenges, particularly in the field of non-cutaneous melanoma.

## 1. Introduction

Since 2010, there have been significant advances in the treatment of melanoma, particularly in the metastatic and high recurrence risk adjuvant settings. These advances have led to the approval of new treatments, such as immunotherapy with anti-PD-1 (pembrolizumab and nivolumab) and anti-CTLA-4 (ipilimumab) antibodies.

Approximately 40% of melanomas harbor the V600 BRAF mutation, leading to constitutive activation of the MAPK signaling pathway. Dual inhibition of this pathway in patients with unresectable V600 BRAF-mutated melanoma, using combination therapy with BRAF and MEK inhibitors, confers high response rates and survival benefit, although efficacy, in metastatic patients, is often limited by development of resistance. Three combinations of targeted therapy with BRAF/MEK inhibitors have received FDA approval in the unresectable setting (dabrafenib and trametinib; vemurafenib and cobimetinib; encorafenib and binimetinib), while dabrafenib and trametinib are also approved as adjuvant therapy following the resection of stage III/IV melanoma. The oncolytic herpes virus talimogene laherparepvec (T-VEC) is also FDA approved for the local treatment of unresectable cutaneous, subcutaneous, and nodal lesions in patients with melanoma recurrent after initial surgery.

In this manuscript, we review melanoma-related clinical trial data, with a focus on data presented or published in 2020. Immune checkpoint inhibitors and targeted therapy continue to dominate the management of melanoma in the metastatic and adjuvant setting. In the metastatic setting, updates on the clinical trials, which have led to the approval of immune checkpoint inhibitors and targeted therapy, show the expected plateau effect in overall survival, with the majority of responders remaining in remission after the completion of treatment. In 2020, the first triple-therapy combining BRAF/MEK targeted and anti-PD-1 immunotherapy with atezolizumab, vemurafenib and cobimetinib was approved for unresectable or metastatic BRAF V600-mutated melanoma (IMspire150 study), while another trial investigating triple therapy missed its endpoint. In the adjuvant setting, updated data continue to support the use of either anti-PD-1 antibodies and BRAF/MEK inhibitors.

However, the clinical decision about when to start treatment, and in what setting, will likely be complicated in the near future with the growing evidence that neoadjuvant therapy is also associated with high rates of recurrence-free survival. Clinical trials comparing adjuvant and neoadjuvant approaches are critical to answer this question. However, these trials to answer the question whether we should treat now or later are lengthy and complex to conduct, especially in an ever-shifting therapy environment (NCT02437279, NCT02977052, NCT02519322, NCT04495010, NCT04013854, NCT03698019). BRAF wildtype patients with primary resistance to checkpoint inhibitors have a particularly dismal prognosis. For these, new therapy options need to be investigated, instead of focusing more trials on the first-line patients with favorable prognostic markers: low lactate dehydrogenase (LDH), no brain metastases, and ECOG performance status of 0 or 1. Finally, other aspects, including the management after resistance and the discovery of new targets, are gaining momentum as research topics of interest in the scientific community.

## 2. Methods

We first started this manuscript by reviewing updates from human clinical trials for metastatic melanoma that were presented at the 2020 ASCO (American Society of Clinical Oncology) Virtual Scientific Program. Our research included the following searches: SessionTitle: “Melanoma/Skin Cancers” AND SessionType: “Poster Discussion Session” AND Meeting: “2020 ASCO Virtual Scientific Program” AND SessionTitle: “Melanoma/Skin Cancers” AND SessionType: “Oral Abstract Session” AND Meeting: “2020 ASCO Virtual Scientific Program.” Further abstracts were found by searching for the term “melanoma” only, and were selected based on relevance and novelty with respect to the purpose of this manuscript. Due to the lack of effective treatments for uveal melanoma, and scarce updates from ongoing clinical trials in the year 2020 for this patient population, we performed a search on ClinicalTrials.gov for the term “uveal melanoma”, and filtered for trials in phases 1, 2 or 3 that were recruiting (as of December 2020); therefore eliminating trials not yet recruiting; or that were suspended, completed, terminated, withdrawn; or that were enrolling by invitation only; or that were active but not recruiting; or that had an unknown status. Regarding study type, we filtered for interventional studies (clinical trials). This search resulted in 27 clinical trials. From those, 7 trials were not specific for uveal melanoma, and another 4 trials addressed only localized disease (localized primary tumor). This was out of the scope of this review, therefore those trials were not discussed. All of the remaining trials (all that addressed metastatic disease) are addressed in our manuscript.

## 3. Metastatic Setting

### 3.1. Update on Efficacy of Checkpoint Therapies

Updated long-term data of the clinical trials assessing efficacy of anti-PD-1 or anti-CTLA-4 monotherapy, or combined pathway blockade for unresectable metastatic melanoma were presented at the 2020 ASCO (American Society of Clinical Oncology) Virtual Scientific Program. The evidence further consolidates the long-term beneficial impact and safety of these agents. Merck’s KEYNOTE-006, a phase 3 trial evaluating two different dosing schedules of pembrolizumab (10 mg/kg IV Q2W or Q3W) compared to ipilimumab (3 mg/kg), underlined that both pembrolizumab regimens had a superior overall survival (OS) compared to ipilimumab monotherapy (three-year follow-up after two years of treatment). Notably, all patients who achieved complete response (CR) were still alive at 5 years [1]. The median OS was 38.7 months for patients treated with first-line pembrolizumab, 23.5 months for second-line pembrolizumab, 17.1 months for first-line ipilimumab, and 13.6 months for second-line ipilimumab [1]. The median OS for first-line pembrolizumab was identical to last year’s outcomes published from the five-year CheckMate 067 trial: the median OS for nivolumab 3 mg/kg was 36.9 months [2]. This trial also reinforced the efficacy of a dual checkpoint blockade: in the nivolumab-plus-ipilimumab group (nivolumab 1 mg/kg and ipilimumab 3 mg/kg Q3W), the median OS was not reached at a five-year follow-up with 52% of patients alive. The 5-year overall response rates of these trials are included in Table 1.

The standard induction phase of a dual checkpoint blockade consists of four doses of ipilimumab and nivolumab. However, real-world data have suggested that patients treated with fewer than four cycles due to toxicity can achieve durable benefits. A phase 2 trial evaluated 60 patients for an early favorable anti-tumor effect (by means of a CT scan at week six) and transitioned responders to maintenance nivolumab instead of the two final cycles of ipilimumab + nivolumab from the standard induction phase [5]. Interestingly, among the patients without early favorable anti-tumor effect at week six, and therefore not selected to de-escalate therapy after the second dose, none ultimately responded to continued combination treatment, suggesting that early identification of non-responders might be possible. This was also proposed to be possible with serological markers [6]. Although early staging may identify non-responders allowing for earlier transition to other therapies and limiting treatment toxicity risks, randomized studies with long-term endpoints are necessary to confirm this and determine whether fewer than four cycles of ipilimumab and nivolumab (before transition to nivolumab monotherapy) can have the same longstanding benefit of the standard regimen. Real world data assessing the survival of melanoma patients comparing first- and second-line therapy suggested that, in second-line or higher, BRAF plus MEK inhibition was superior to anti-PD-1 monotherapy throughout the first three years [7].

### 3.2. Targeted Therapies

Regarding BRAF targeted therapy, an update of the COLUMBUS trial, with a median five-year follow-up across all arms, revealed a median OS of 33.6 months for encorafenib + binimetinib, compared to 23.5 and 16.9 for monotherapy with encorafenib and vemurafenib, respectively, confirming the use of dual MAPK pathway blockage as opposed to inhibition of BRAF alone [8].

In patients with V600 BRAF mutated melanoma, optimal treatment sequencing is unknown given the lack of randomized, outcome data from studies systematically sequencing the order of anti-PD-1 and anti-BRAF targeted treatment. However, due to alternative and potentially synergistic mechanisms of action, investigation explored triple therapy with concurrent anti-PD1/PD-L1 and BRAF and MEK inhibitor-based treatment. These studies have demonstrated mixed results. The IMspire150 study randomized 514 treatment-naïve patients with V600 BRAF mutated unresectable stage III/IV melanoma to treatment with vemurafenib and cobimetinib (V/C) plus or minus the PD-L1 inhibitor atezolizumab. The run-in phase of V/C extended over four weeks, and atezolizumab was then added to the scheme. The primary endpoint of investigator-assessed progression-free survival (PFS) demonstrated a significant improvement of 4.5 months difference with the addition of atezolizumab (15.1 vs. 10.6 months) [9]. This led to FDA approval of this triple therapy for treatment of this patient population. Toxicity was remarkable, with 79% experiencing severe side effects and treatment discontinuation in 45%. The most common grade 3–4 adverse events were rash, musculoskeletal pain, fatigue, hepatotoxicity and pyrexia. There were a total of 14 therapy-induced fatalities in the trial (seven in each group, corresponding to 3% in the triple-therapy group and 2.5% in the V/C + placebo group). Atezolizumab is the first anti-PD-L1 antibody approved to treat metastatic melanoma. This trial also reported other endpoints, such as incidence and time to development of brain metastases with first-line combination treatment with V/C combined with atezolizumab or placebo [10]. After a median follow-up of 18 months, 21% of the patients receiving triple therapy had developed brain metastases, compared to 25% in the control group with only V/C. Prospective trial data of how the efficacy of first-line triple therapy compares to upfront treatment with ipilimumab plus nivolumab are still missing.

After six weeks of pembrolizumab treatment, patients with advanced melanoma expressing BRAF V600E/K were randomized, as part of the phase 2 IMPemBra trial, to continue pembrolizumab alone or added dabrafenib plus trametinib (two cohorts of intermittent dosing and a cohort of continuous dosing for six weeks). Continuous targeted therapy led to a 62% rate of grade 3 or higher treatment-related adverse events, with the majority of patients needing dose interruption or discontinuation, particularly due to fever and hepatitis. Only 38% of patients treated with continuous dabrafenib + trametinib (D/T) received all planned treatment, as opposed to 88% of patients treated with intermittent D/T for 2 × 1 week, and 63% of patients treated with intermittent D/T for 2 × 2 weeks. The median progression-free survival (PFS) of patients treated with pembrolizumab monotherapy was 10.6 months, which nonsignificantly increased by 16.4 months to 27.0 months for patients treated with pembrolizumab plus intermittent, short-term, dabrafenib and trametinib (*p* = 0.13) [11]. Long-term follow-ups and larger studies are required to validate these results and to determine whether this combination translates into an overall survival benefit.

The randomized phase 3 COMBI-i trial (dabrafenib plus trametinib ± the PD-1 inhibitor spartalizumab) demonstrated high objective response rates (ORR) and CR rates (78% and 44%, respectively), with triple therapy and a high frequency of durable responses (two-year OS of 74%), including in patients with poor prognostic factors (ORR in patients with elevated LDH of 67%). However, the trial failed to meet its primary endpoint of improved investigator-assessed PFS. Grade 3–5 adverse events were detected in 53% of patients, leading to the discontinuation of treatment in 17% [12].

The LOGIC2 phase 2 study aims to evaluate the benefit of a third agent selected at progression, based on the genetic tumor evolution assessed by next generation sequencing in addition to encorafenib/binimetinib [13]. One of four agents with distinct mechanisms is being studied: a CDK4/6 inhibitor, a PI3K inhibitor, a c-Met inhibitor and a Fibroblast Growth Factor Receptor (FGFR) inhibitor. In an initial report, safety was found to be consistent with known profiles of the individual agents, however the increased anti-tumoral activity was minimal (for the first three cohorts: ORR 5.3%, 0% and 0%, respectively; median PFS of 2.1, 1.6, and 2.2 months). This study resembles the NCT MASTER trial which found that DNA and RNA analyses support treatment recommendations in 10% to 57% of patients.

### 3.3. Resistance to Checkpoint Therapy

Despite the striking impact of anti-PD-1 and anti-CTLA-4 drugs on the survival rates of melanoma patients, several problems arise from these treatments. Firstly, there are patients who unfortunately do not respond (primary resistance), and others who stop responding after an initial response (acquired resistance). The approach to anti-PD-1 resistance is one of the most pressing problems in the treatment of melanoma, and it is one of the most explored topics of the last few years. The efficacy of anti-CTLA4 monotherapy versus combined anti-CTLA-4 and anti-PD-1 therapy has not been well defined in melanoma patients who progressed on anti-PD-1 monotherapy. A multicenter study with 300 patients (44% of which were BRAF mutated) evaluated the management of patients who achieved CR or partial response (PR) to anti-PD-1 therapy, and who later progressed [14]. Acquired resistance was found to be oligometastatic, occurring in the form of a new lesion in 45.3% of the patients, in existing lesions in 35.3%, and both in new and existing lesions in 19.3%. For patients who received systemic treatment after acquired resistance, anti-PD-1 was the most common choice (51% of the patients; 41% continuation, 59% reinduction) compared to dual checkpoint inhibition with anti-PD-1 and anti-CTLA-4 in 12%; anti-CTLA-4 monotherapy in 6%; targeted therapy in 19%; and investigational drugs in 11%. The objective response rate (ORR) to subsequent treatment was 46% for PD-1 monotherapy (56% continuation, 42% reinduction), 56% for PD-1 + CTLA-4, 0% for CTLA-4 alone, 7% for targeted therapy and 20% for investigational drugs. With a median follow-up from acquired resistance of 20 months, there was no difference in OS by systemic treatment class.

Another retrospective, multicenter trial with 330 patients assessed response rate and survival of advanced melanoma patients resistant to anti-PD-1 therapy (70% primary, 30% acquired) following treatment with either ipilimumab monotherapy or ipilimumab in combination with anti-PD-1 antibodies [15]. With a median follow-up of 22.3 months from the start of ipilimumab, a higher response rate was seen with the dual therapy (31% vs. 13%), as was one-year PFS (27% vs. 13%). Therefore, in the setting of anti-PD-1 resistance, the use of a combined CTLA-4 and PD-1 blockade may confer an improved chance of response and longer PFS when compared to treatment with anti-CTLA-4 monotherapy.

The results of the first prospective clinical trial evaluating ipilimumab 1 mg/kg + pembrolizumab 200 mg following progression on PD-1 blockade [16] displayed a response rate of 30% and PFS at six months of 45%, highlighting dual checkpoint blockade as an option following progression on anti-PD-1 agents. As expected [17], tolerability was good, with 12% of patients experiencing ≥grade 3–4 immune-related adverse events (irAEs) as compared to the higher rate seen with ipilimumab dosed at 3 mg/kg with lower doses of anti-PD-1. PD-L1 tissue expression did not associate with response, as is generally the case for melanoma.

Lastly, a multicenter analysis focused on the management of recurrence after adjuvant targeted therapy [18]. Patients who developed recurrent melanoma during and after cessation of adjuvant targeted therapy were included (*n* = 87). A large number of patients (86%) received systemic therapy: 46% anti-PD-1 based therapy, i.e., anti-PD-1 monotherapy or combined with anti-CTLA-4 or an investigational drug; 14% ipilimumab monotherapy, 21% retreatment with combination BRAF/MEK inhibitors; and 6% other agents (e.g., chemotherapy, TVEC). The response rate after relapse was assessed in 33 patients, demonstrating a three-year OS of 79% for anti-PD-1 based therapy, 55% for targeted therapy, and 25% for ipilimumab, suggesting that patients who relapse after adjuvant targeted therapy may respond to a subsequent checkpoint blockade at similar rates to the treatment-naïve setting. A current ongoing multicenter study is investigating the effect of short-term chemotherapy followed by a re-challenge of checkpoint inhibitors in patients with primary resistance to checkpoint inhibitor therapy (NCT04225390).

### 3.4. Toxicity and Checkpoint Therapy

Another challenge of the current landscape of treatments available for melanoma patients is high grade toxicity with rates of 55–59%, 20%, and 27% induced by treatment with ipilimumab plus nivolumab, nivolumab alone, and ipilimumab alone, respectively. A retrospective study with 626 melanoma patients treated with anti-PD-1 monotherapy aimed to characterize differences in the response and irAEs patterns between different ethnic groups [19]. Caucasians had significantly higher ORRs, but this did not translate into a survival advantage. Distinct irAE patterns were also observed between ethnic groups, e.g., lower incidence of most irAEs in Caucasians. Pneumonitis, however, was remarkably more frequent in Caucasians. Endocrine, musculoskeletal, and skin irAEs were associated with improved PFS and OS across ethnicities.

Many of the trials mentioned in previous sections of this manuscript have been presented at scientific conferences and have seen abstracts published; however, a comprehensive analysis of the safety profiles must be performed once the complete data have been published in peer-reviewed journals.

### 3.5. Novel Therapies in Late Stage Disease

Despite many breakthroughs in basic research, few new strategies have successfully translated into late-stage clinical trials. Lifileucel represents a promising approach that utilizes lymphodepletion followed by the autologous adoptive cell transfer of tumor-infiltrating lymphocytes and six doses of high-dose interleukin-2. Long-term follow-up data of 66 heavily pretreated melanoma patients subsequently treated with lifileucel, demonstrated an ORR, as assessed by investigators, of 36.4% (2 CR, 22 PR), and a disease control rate of 80.3%, with a median duration of response not reached at 17 months follow-up [20].

A trial with 58 patients, including 34 melanoma patients, investigated the use of AZD6738, an oral inhibitor of the serine/threonine protein kinase ATR (Ataxia Telangiectasia and Rad3 Related) to treat refractory advanced solid tumors in combination with weekly paclitaxel. The authors report one complete response and 12 partial responses in 51 patients eligible for evaluation of efficacy (ORR of 25.5%) [21].

Another phase 1 study evaluating a new drug in combination with chemotherapy used 9-ING-41, an inhibitor of GSK-3β, a serine/threonine kinase, as a single-agent or combined with different chemotherapy schemes. Aside from one complete response in a BRAF-mutated melanoma patient with brain metastases, only 8 of 101 patients remained on the trial longer than five months. A biologically active dose of 9-ING-41 has been reached, the maximum tolerated dose was not determined, and enrollment is still ongoing [22].

A first-in-human trial (*n* = 34) of CX-2029, an antibody–drug conjugate therapeutic directed against CD71, reported AEs in 88% of the patients, from which the most common were hematological events. From 32 response-evaluable patients, the authors report one partial response and nine patients with stable disease, including one ocular melanoma patients treated for 36 weeks [23].

Neurotrophic tropomyosin receptor kinase (NTRK) fusions can be found in melanoma, and tropomyosin receptor kinase (TRK) inhibitors larotrectinib and entrectinib are approved to treat solid tumors with this key genetic driver, therefore there are clinical trials to investigate these drugs in melanoma patients (NCT02465060, NCT02576431, NCT02568267).

In the field of cancer vaccines, a phase 2 trial with a liposomal RNA vaccine targeting four non-mutant shared tumor-associated antigens (MAGE-A3, NY-ESO-1, tyrosinase, TPTE) showed encouraging results in melanoma patients who had progressed on checkpoint therapy. In the vaccine monotherapy cohort, 12% of patients experienced a partial response; 35% in the combination cohort (vaccine + anti-PD-1) [24].

As treatment options expand, it is essential to identify biomarkers of efficacy. A retrospective multicenter study with 101 patients, including, but not limited to, melanoma patients, has suggested better outcomes to immune checkpoint blockade for patients with alterations in the tumor suppressor gene LRP1b (low-density lipoprotein receptor-related protein 1b). Patients with pathogenic LRP1b alterations had better ORRs and PFS—even after excluding microsatellite instability (MSI)-high or tumor mutational burden (TMB) tumors—compared to patients with variants of undetermined significance alterations [25].

Other upcoming treatment modalities in clinical trials include quadruple therapy with BRAK/MEK inhibitors in combination or sequential regimens with anti-CTLA-4 and anti-PD1/PD-L1 antibodies (NCT01940809, NCT02968303, NCT02224781); intratumoral RNA-based TLR-7/-8 and RIG-I agonists as monotherapy or in combination with anti-PD-1 drugs (NCT03291002); subcutaneous and intratumoral DNA TLR-9 agonists in monotherapy (NCT04126876) or combination with checkpoint inhibitors (NCT03445533, NCT04401995, NCT03618641); T cell redirection with tebentafusp (formerly known as IMCgp100) as monotherapy or combined with anti-CTLA-4 antibody tremelimumab, or anti-PD-L1 antibody durvalumab, or both (NCT02535078); histone deacetylase (HDAC) inhibitors (e.g., entinostat, panobinostat, abexinostat) in combination with ipilimumab (NCT02032810) or anti-PD-1 (NCT02697630, NCT03765229, NCT03590054); and intratumorally administered stimulator of interferon genes (STING) agonist (NCT04144140).

## 4. Adjuvant Setting

The three-year follow-up of pembrolizumab’s adjuvant trial Keynote 054 displayed improved recurrence-free survival (RFS) across all subgroups (stages IIIA, IIIB and IIIC; PD-L1 positive and negative; BRAF-mutated and wildtype) [26]. In the overall population analysis, the pembrolizumab-treated group had a three-year RFS of 64% compared to 44% in the placebo group. Notably, these values were 81% and 66% in stage IIIA patients.

Nivolumab has been shown to have recurrence-free survival benefit when compared to ipilimumab in resected stage IIIB-C or IV melanoma, although the results were identical in regard to overall survival at four years (77.9% for nivolumab and 76.6% for ipilimumab), raising the important question of whether benefits in recurrence-free survival translate to significant improvement of overall survival in the modern era of systemic therapies [27].

The five-year analysis of adjuvant dabrafenib and trametinib in patients with resected stage III BRAF V600-mutant melanoma confirmed the long-term benefit of the treatment with median RFS not being reached for dabrafenib and trametinib vs. 16.6 months in the placebo arm [28]. The five-year RFS rates were 52% vs. 36%, respectively (Table 2).

A multicenter analysis of 87 patients with melanoma recurrent after treatment with adjuvant targeted therapy (27% during and 76% following; median time to first recurrence of 16.3 months) demonstrated a high response rate to anti-PD-1 therapy, suggesting that subsequent response to immunotherapy remains similar to the treatment-naïve setting [18]. However, in terms of choosing the modality of adjuvant therapy, randomized data comparing adjuvant dabrafenib plus trametinib to anti-PD-1 therapy remain lacking. Thus, toxicity profiles can guide the choice of therapy in this setting with, for example, life-altering adverse events in immunotherapy but not in targeted therapy.

Updates from these clinical trials, and others, are expected in future. The safety profiles must be carefully discussed and compared to the data from trials in the metastatic setting.

With regard to cancer vaccines, one phase 3 trial with seviprotimut-L—an allogeneic, polyvalent vaccine derived from three human melanoma cell lines, that was given to stage IIB-III melanoma patients—was not positive in the intent-to-treat population. However, patients with stage IIB/IIC (particularly those younger than 60 and with ulcerated primary melanoma) had longer recurrence-free survival [29].

## 5. Neoadjuvant Setting

There is still no approved neoadjuvant regimen for melanoma. However, growing evidence demonstrates feasibility. The two-year follow-up data from a phase 2 neoadjuvant trial assessing three different dosing schedules of ipilimumab + nivolumab has been presented [30]. The trial included the following arms: two courses of 3 mg/kg ipilimumab + 1 mg/kg nivolumab Q3W (arm A); two courses of 1 mg/kg ipilimumab + 3 mg/kg nivolumab Q3W (arm B); two courses of ipilimumab 3 mg/kg, directly followed (>2 h and <24 h) by two courses of nivolumab 3 mg/kg Q2W (arm C). RFS and event-free survival (EFS) did not differ between the arms. Out of 86 patients, five patients died (four due to melanoma and one due to toxicity, i.e., late-onset immune-related encephalitis [31]); 55 patients (68%) reported ongoing irAEs.

Notably, no difference between treatment arms was observed regarding the prevalence of irAEs. The authors conclude that these outcomes support comparing two cycles of neoadjuvant ipilimumab (1 mg/kg) + nivolumab (3 mg/kg) versus adjuvant anti-PD-1 in a randomized phase 3 trial.

An experimental extension cohort (PRADO; *n* = 99) of this trial reported that therapeutic lymph node dissection was omitted in 97% of the patients who achieved a complete or near-complete pathological response (≤10% viable tumor cells), therefore reducing surgical morbidity [32]. The previously reported high-grade toxicity of this trial varied between arms: grade 3–4 adverse events were seen in 40% of patients in arm A, 20% in arm B, and 50% in arm C. The most common grade 3–4 adverse events were elevated liver enzymes in group A (20%) and colitis in group C (19%); in group B, none of the grade 3–4 adverse events were seen in more than one patient.

There is great expectation on how neoadjuvant treatment will affect the management of patients with melanoma, not only due to direct consequences regarding surgery, but also because of its impact on adjuvant treatment and for the management after progression. Currently, adjuvant treatment with anti-PD-1 and targeted agents is approved for melanoma patients with involvement of lymph nodes (stage III) or metastatic disease (stage IV) who have undergone resection. If neoadjuvant treatment is approved, there will be no immediately available information on whether to continue treatment post-surgery (in an adjuvant setting), because neoadjuvant trials do not contain such an arm. Furthermore, one can speculate that management after recurrence in patients who received neoadjuvant therapy will be similar to that being investigated in other settings (adjuvant and metastatic), but clinical trials will be necessary to determine this. Moreover, data from trials comparing neoadjuvant versus adjuvant treatment to fully determine the optimal approach to resectable metastatic melanoma are still missing.

## 6. Non-Cutaneous Melanoma

Mucosal melanoma and uveal melanoma have lower response rates to anti-PD-1 and anti-CTLA-4 inhibitor immunotherapy when compared to cutaneous melanoma.

A subgroup analysis of patients with mucosal melanoma treated within the CheckMate 067 trial with first-line treatment with ipilimumab, nivolumab, or combined therapy with ipilimumab + nivolumab was reported. Five-year outcomes of mucosal melanoma patients (*n* = 79) showed similar safety profiles, but poorer long-term efficacy compared to the intent-to-treat population (ORR of 43% for ipilimumab + nivolumab vs. 30% for nivolumab monotherapy vs. 7% for ipilimumab monotherapy; PFS of 29%, 14% and 0%, respectively; OS 22.7, 20.2 and 12.1 months) [33]. This highlights the need for new agents for this type of melanoma, and it also supports preference for dual checkpoint therapy when treating mucosal melanoma. A phase 1b trial with a combination of VEGF inhibition (axitinib) with PD-1 blockade (toripalimab) had an ORR of 48.3%, median PFS of 7.5 months, and median OS of 20.7 months in 29 treatment-naïve mucosal melanoma patients [34]. A randomized three-arm phase 2 trial to compare toripalimab plus axitinib with toripalimab or axitinib monotherapy has been initiated.

In regard to uveal melanoma, a number of clinical trials have failed to add benefit to this patient population. These included the use of adjuvant crizotinib in patients with high recurrence risk disease [35], and ERK inhibition with ulixertinib [36]. Additionally, there is still discussion about the best approach for liver metastases, the most common site of metastasis in those patients [37,38]. Ongoing clinical trials that address this question include combinations of immunoembolization with checkpoint inhibitors (NCT03472586), hepatic perfusion as monotherapy (NCT01785316) or combined with checkpoint blockade (NCT04283890), transarterial radioembolisation and transarterial chemoembolisation (NCT02936388), and PV-10 chemoablation (NCT00986661).

Promising clinical approaches for uveal melanoma in the adjuvant setting include adjuvant dendritic cell (DC) vaccination with autologous tumor RNA (NCT01983748), and sunitinib or valproic acid (NCT02068586). In patients with metastatic disease, currently recruiting clinical trials include novel immunotherapies such as tebentafusp (a first-in-class bispecific fusion protein that targets the melanoma-associated antigen gp100, NCT03070392), adoptive cell therapy (NCT03467516, NCT03467516, NCT03068624), combinations of radiation therapy and immunotherapy agents (NCT02913417), combinations of checkpoint inhibitors with novel molecules such as relatlimab (NCT04552223), MEK inhibitor with the FAK inhibitor IN10018 (NCT04109456), RO7293583 (a putative CD3 T-Cell engager targeting TYRP1, + tocilizumab +/− obinutuzumab, NCT04551352), intermittent therapy with selumetinib (NCT02768766), and DC vaccination in combination with a dual checkpoint blockade in metastatic patients (NCT04335890). In this last setting, DC vaccination has been shown to be safe and to induce immunological and clinical responses, including a median overall survival of 36.4 months [39]. A phase 2 trial has shown an ORR of 18% for a regimen of nivolumab plus ipilimumab in patients with metastatic uveal melanoma (*n* = 33), a median PFS of 5.5 months, and median OS of 19.1 months [40], further highlighting the need for the development of novel therapies for this population.

Lastly, a single-center phase I/Ib study of concurrent intrathecal and intravenous nivolumab (N) for metastatic melanoma with leptomeningeal disease (*n* = 15) was also presented at ASCO [41]. Notably, there were no grade 4–5 irAEs and no grade 3–5 AEs attributed to the intrathecal administration. With a median follow-up of 18.7 weeks (range 1–83.3), the median OS was 46.1 weeks.

## 7. Conclusions

In conclusion, advances in dermato-oncology have led to a five-year survival rate of about 50% in patients with cutaneous metastatic melanoma. The optimal sequencing of therapy options in BRAF-mutated patients remains to be determined. Additionally, the move towards treating patients at earlier stages has started. In this setting, the analysis of potentially life-changing adverse events is even more important. Patients with primary resistance to checkpoint inhibitors and difficult to treat subgroups, such as mucosal and uveal melanoma, still greatly need innovative treatment approaches to improve their prognosis.

## Figures and Tables

**Table 1:** 5-year overall survival (OS) rates for approved therapy regimens in patients with metastatic cutaneous melanoma.

	Approved Drug or Combination	5-Year OS Rate	Reference
Checkpoint inhibitors	Pembrolizumab (first-line)	43.3%	[1]
	Nivolumab (first-line)	44%	[2]
	Ipilimumab (first-line)	26–33%	[1,2]
	Ipilimumab + nivolumab (first-line)	52%	[2]
Targeted therapy	Dabrafenib + trametinib	34%	[3]
	Vemurafenib + cobimetinib	39.2%	[4]
	Encorafenib + binimetinib	Not yet available (only 3-year outcomes published thus far)	

**Table 2:** Long-term recurrence-free survival (RFS) rates for adjuvant therapy regimens in patients with metastatic cutaneous melanoma.

	Available Long-Term Outcomes	Drug or Combination	RFS Rate	Reference
Targeted therapy	Outcomes only available for one combination: 5-year outcomes for D+T	Dabrafenib + trametinib (D+T)	52%	[28]
Checkpoint inhibitors	5-year outcomes not yet published, only 3-year (P) and 4-year (N, I) outcomes available	Pembrolizumab (P)	64%	[26]
		Nivolumab (N)	51.7%	[27]
		Ipilimumab (I)	41.2%	[27]
